# Level of Physical Activity and Its Relationship to Self-Perceived Physical Fitness in Peruvian Adolescents

**DOI:** 10.3390/ijerph19031182

**Published:** 2022-01-21

**Authors:** Roxana Paola Palacios-Cartagena, Jose A. Parraca, Maria Mendoza-Muñoz, Raquel Pastor-Cisneros, Laura Muñoz-Bermejo, Jose Carmelo Adsuar

**Affiliations:** 1 Promoting a Healthy Society Research Group (PHeSO), Faculty of Sport Sciences, University of Extremadura, 10003 Cáceres, Spain; ropalacio@alumnos.unex.es (R.P.P.-C.); mamendozam@unex.es (M.M.-M.); raquelpc@unex.es (R.P.-C.); jadssal@unex.es (J.C.A.); 2 Departamento de Desporto e Saúde, Escola de Saúde e Desenvolvimento Humano, Universidade de Évora, 7000-654 Évora, Portugal; 3 Comprehensive Health Research Centre (CHRC), Universidade de Évora, 7000-654 Évora, Portugal; 4 Social Impact and Innovation in Health (InHEALTH), University of Extremadura, 10003 Cáceres, Spain; lauramunoz@unex.es

**Keywords:** PAQ-A, IFIS, adolescents, physical activity, physical fitness

## Abstract

Background: Physical activity and physical fitness play an important role in adolescence. Both are considered to be indicators of the current and future health status of young adults. The main objective of this article was to report the normative values of the Physical Activity Questionnaire for Adolescents (PAQ-A) and the International Fitness Scale (IFIS) instruments in Peruvian school adolescents. Methods: A sample of 1229 participants (622 girls and 607 boys) aged between 12 and 17 years was used. The type of study was descriptive-comparative. All measures used were obtained by means of self-administered instruments. The PAQ-A was used to assess the level of physical activity and the IFIS to assess the self-perceived physical fitness level of the adolescents. Results: It was observed that the PAQ-A questionnaire results obtained from the total sample was 2.34; significantly higher for boys (2.41) compared with girls (2.27). For the IFIS, the total score was 3.07, with boys obtaining 3.13 and girls 2.97. Conclusions: It was concluded that there was a direct relationship between the level of PA and self-perceived PF in Peruvian adolescents. Furthermore, adolescent boys were more physically active than girls and they had a better self-perceived PF with the exception of flexibility. Finally, there was a higher weight category involved at the lower level of PA.

## 1. Introduction

The level of physical activity (PA) is a decisive component of adolescent health. A continued low level of PA is related to cardiovascular and metabolic problems. PA is one of the behaviours that determine health status as it plays a positive role in preventing morbidity and mortality in individuals [1]. For this reason, it is important to promote healthy habits based on exercise to prevent sedentary behaviour [2]. Currently, a minimum of 60 minutes of moderate or vigorous intensity PA per day is recommended for adolescents, as set by the World Health Organisation (WHO) [3].

Physical fitness (PF) is the ability to perform energetic activities and is directly related to health. It involves cardiorespiratory endurance, muscular strength, motor skills and body composition [4]. Several scientific studies recognise that PF in adolescence is a reference guide to the current and future health status of a person [4,5]. Furthermore, people who practice PA on a regular basis report better self-perceptions of their PF than those who undertake it less frequently [6]. Numerous studies have evaluated physical inactivity in children and young people, reporting similar and worrying results [7,8,9]. In this regard, the WHO indicates that more than 80% of adolescents in school (85% of girls and 78% of boys) do not reach the recommended minimum level of one hour of physical activity per day worldwide [3,9].

Physical fitness in childhood and adolescence is considered to be a predictor of mortality and comorbidities resulting from physical inactivity [10,11]. Thus, assessing physical fitness during the school years may be crucial for the prevention or early detection of pathologies associated with excess body fat. Physical fitness assessments provide information on the physical condition of a subject as well as the effects of physical activity practices on it [12], which might help to make decisions about the most appropriate physical activity guidelines.

In order to detect the levels of PA and self-perceived PF in the adolescent population, it is essential that we have effective tools. There exist numerous objective ways for assessing physical fitness in children and adolescents, including test batteries such as the Assessing Levels of Physical Activity (ALPHA-Fitness) [13], the European Physical Fitness battery (EUROFIT) [14] or the Assessment of Health-Related Physical Fitness (COFISA) [15]. However, the subjective component based on the evaluation of self-perceived physical fitness should be also considered. In this regard, the International Fitness Scale (IFIS) instrument has been applied in different contexts to analyse self-perceived PF [16,17,18]. Regarding self-perception of PA, the Physical Activity Questionnaire for Adolescents (PAQ-A) instrument has been considered, which has been recognised as an effective and reliable measure of the overall level of PA from childhood to adolescence [19,20].

The levels of PA and PF can be objective and accurate if assessed from laboratory or field tests. However, this is costly in the case of the former and time-consuming in the case of the latter, so the use of these assessments may be somewhat limited. An alternative method that could be used to assess both are self-perception surveys. In this sense, both the PAQ-A and the IFIS are cost-effective, time-efficient and easy-to-complete instruments. In addition to providing information, they provide an insight into the self-perceived health status of an adolescent [16,18].

There are several studies linking current fitness assessed by field tests and self-perceived fitness. In [21], a high correlation between general fitness and general physical self-concept factors (r = 0.76) was shown. Similarly, the study by Jakkola and Washington [22] showed a correlation between perceived fitness and actual physical activity. There are also several authors who have correlated endurance field tests with self-perceived endurance ((r = 0.50) [23], (r = 0.45) [24], (r = 0.27) and (r = 0.33 to r = 0.53) [25]) as well as a strength field test with self-perceived strength (between r = 0.14 to r = 0.18 [26], r = 0.18 [27] and r = 0.09 [28]); likewise from a speed field test with self-perceived speed (between r = 0.38 to r = 0.56 [29], r = 0.75 [30] and r = 0.39 [22]). There are also flexibility field tests that relate to self-perceived flexibility ((r = 0.53) [31], (r = 0.04) [32], (r = 0.21) [21] and (r = 0.15) [33]).

In Peru, there are few studies on the subject; unfortunately, Peruvian adolescents currently have insufficient and limited PA, which is reflected in sedentary lifestyles. Only one in three Peruvians over the age of fifteen is physically active, a condition that represents a risk factor for chronic diseases. This also indicates that most Peruvians do not comply with the recommendations indicated for daily physical activity [34]. However, in the study conducted by Bustamante [35] with Peruvian adolescent children, he found an increase in fitness levels with age.

It is worth mentioning that this is the first study in Peru to evaluate the level of PA and its relationship with self-perceived PF in school students as there is no previous evidence. Therefore, the aims of this study are to establish the normative values for the level of PA in Peruvian adolescents, to study the differences between the sexes, to examine their self-perceived PF and to analyse the relationship between the level of PA and self-perceived PF.

## 2. Materials and Methods 

### 2.1. Ethics Approval and Participants

Ethical approval was granted by the Bioethics and Biosafety Committee of the University of Extremadura on 10 December 2020 (approval number: 162/2020) in accordance with the updates of the Declaration of Helsinki as amended by the 64th General Assembly of the World Medical Association (Fortaleza, Brazil, 2013) and the Law 14/2007 on Biomedical Research. This approval included a formal declaration signed by the parents or legal guardians allowing the participation of their children in the present study.

The data collection was carried out with school pupils and took place in school or during after-school sports activities. Using a mobile phone, the students accessed the survey link and completed the questionnaire. All participants met the following inclusion criteria: (1) being between 12 and 17 years of age; (2) informed consent signed by parents or legal guardians; (3) acceptance of the participant in the study.

The total sample consisted of 1229 adolescent students.

### 2.2. Study Design, Instruments, Procedure and Statistical Analyses 

#### 2.2.1. Study Design 

A single-measure descriptive and correlational cross-sectional study was conducted.

#### 2.2.2. Instrument

##### Socio-Demographic Data 

The socio-demographic characteristics collected in the survey were age, sex, weight, height, BMI and educational level. 

##### Level of Physical Activity 

This was assessed on the basis of the PAQ-A questionnaire, which was originally developed in English and aimed at adolescents. The cultural adaptation of the PAQ-A to Spanish was carried out following the basic steps of the standardised procedure for the cultural adaptation of questionnaires [36].

The PAQ-A consists of nine questions that assess different aspects of PA performed by the adolescent such as walking, dancing, playing and jumping among other frequent activities performed during the week. This includes school activities such as a physical education class [19]. The overall result of the test is a score from 1 to 5 up to question 8. Question 9 allows for observations such as if the adolescent was ill or if something prevented him/her from exercising during the week. Its use is valid if it is measured during the school period so it should not be administered during holidays [37]. This questionnaire can be administered during a school class and takes approximately 10–15 min to complete. Our participants completed the Spanish version (ICC = 0.71) [38]. The PAQ-A has previously been used on Spanish adolescents, showing an adequate reliability and validity for assessing physical activity [37]. Likewise, the PAQ-A has been applied to Polish adolescents where the results showed that this instrument is useful in clinical practice and in epidemiological studies to assess general PA levels in adolescents, proving to be reliable and valid [38]. Along the same lines, several studies have shown that the PAQ-A is a reliable and valid questionnaire to be applied to adolescents [39,40,41].

##### Self-Perceived PF

This measure was assessed with the International Fitness Scale (IFIS), which was originally created in English and subsequently translated and validated in several languages, including Spanish [42]. This instrument consists of five Likert-type scale items: general PF; cardiorespiratory fitness; muscle strength perception; speed agility; and flexibility. The response possibilities are very bad, bad, acceptable, good and very good. The estimate for each item is 1–5 [43]. Several studies have shown its reliability and validity in an adolescent population (Kappa = 0.45) [42,44]. Likewise, the IFIS has shown its high validity and good reliability in young adults [16] as well as in children [45].

#### 2.2.3. Procedure

The study was conducted during the period from September 2020 to June 2021 in Peru. During the entire period, the classes were conducted virtually due to the COVID-19 pandemic with the approval of the schools and the parents or legal guardians of the students. All questionnaires were virtually conducted during the physical education class. The students were given the opportunity to complete the questionnaire for a maximum of 40 minutes and almost all of them finished before that time. In addition, the students could ask any questions they had and they were answered instantly. The data collection was carried out using a mobile phone, computer or tablet; the students accessed the survey link and completed the questionnaire in this way. The Google format was used, in which the questions included in this manuscript had to be answered in order to submit the questionnaire responses so there were no unanswered questions. The biggest problems we encountered were in the questions about height as this was asked in centimetres; a few participants gave the information in metres, which we had to convert into centimetres.

### 2.3. Statistical Analyses

All information collected was tabulated in a database designed specifically for this study. The statistical analyses were performed with SPSS software, version 25 (IBM SPSS, Chicago, IL, USA) and personal data were kept anonymous. 

Data were presented as a mean and standard deviation and a median and interquartile range both for the total sample and segmented by sex. The normality and homogeneity were tested using the Kolmogorov–Smirnov test and Levene’s test, respectively. The Mann–Whitney U test was used to establish the sex differences for all variables. Differences were considered significant at *p* ≤ 0.05. To quantify the magnitude of changes between the study groups, the effect sizes were calculated using Cohen’s d [46]. 

Finally, to quantify the relationships between the variables, the Spearman correlation coefficient was applied. The Bonferroni correction was applied from the formula α* = α/n − 1 [47] where α* is the corrected value at which the null hypothesis should be rejected and n is the number of hypothesis pairs. Therefore, the alpha significance level was set at 0.012 for multiple comparisons between the PA level and the self-perception of PF. Correlation values were interpreted following Cohen’s classification thresholds [46]: 0.30 to 0.59, moderate; 0.60 to 0.79, high; and ≥0.80, excellent.

## 3. Results

Table 1 shows the anthropometric characteristics as well as the level of PA and self-perceived PF of Peruvian school students. A total of 1229 adolescents participated in the survey. Of these, 622 were girls and 607 were boys. It could be observed that there were no significant differences with respect to age. On the other hand, it could be observed that, in the variables of height and weight, the boys showed significantly higher values than the girls. In addition, the girls had a significantly higher BMI than the boys. With respect to the level of PA, it was observed that in the PAQ-A questionnaire, the boys (2.41) showed significantly higher values than the girls (2.27).

In terms of self-perception of PF, the boys scored significantly higher than the girls for all items of the IFIS scale with the exception of the flexibility item where there were no differences. In addition, it was observed that in general physical fitness, the boys showed a high percentage in the “acceptable” option (61.4) compared with the girls (58.6). In cardiorespiratory fitness, the highest value was obtained in the “acceptable” option where the girls obtained 60.7%, slightly higher than the boys (59.9%). In the dimension of muscular strength, the highest score was observed in the “acceptable” option for the boys with 38.9% whereas the girls indicated 35.5%. In the speed dimension, the boys showed a similar percentage compared with the girls (42.9% and 42.7%, respectively). Finally, in the flexibility dimension, we observed that the highest percentage was in the “acceptable” option with 61.6% for the girls and 60.7% for the boys, highlighting that this dimension was the highest of all.

Table 2 presents the normative values for the PA level of Peruvian adolescents, differentiated by sex and segmented by age and BMI category. It could be seen that there was a statistically significant difference between the sexes in the level of PA for ages 12, 13, 15 and 17 years, with the boys having a significantly higher level of PA than the girls. The highest PA level was found in the boys aged 15 years with a mean score of 2.51 compared with the girls, which was found in the age group of 16 years with a mean score of 2.35. On the other hand, the lowest level of PA was found at the ages of 14 and 17 years in the girls with a mean score of 2.22 and in the boys at 14 years with a mean score of 2.30. With regard to the BMI, it was observed in both sexes that the higher the weight category, the lower the PA level and, consequently, the lower the PAQ-A score. In addition, a significantly higher PA level was indicated in the boys than in the girls for all weight categories except the underweight category.

In Table 3, the correlation between the PA level and self-perception of PF level can be seen. A high direct correlation was found between the PA level (PAQ-A) and all dimensions of self-perceived PF (IFIS) (r = 0.644 to r = 0.604) with the exception of the flexibility dimension whose correlation was moderate (r = 0.404). When segmented by gender, this correlation prevailed, being higher in the girls (r = 0.692 to r = 0.466) than in the boys (r = 0.594 to r = 0.336).

## 4. Discussion

This study analysed the normative values of PA level in Peruvian adolescents, the differences between sexes and their self-perceived PF as well as the relationship between the two. 

The main findings of this study indicates that boys scored higher on the PA level than girls. Regarding self-perceived PF, the results obtained indicated that boys achieved better self-perceived levels in four dimensions (PF, muscular strength, cardiorespiratory fitness and speed) whereas girls only achieved better self-perception in the level of flexibility.

Regarding the level of PA, it is worth mentioning that the availability of normative data in the adolescent population is fundamental for making comparisons, diagnoses, interventions, treatments and evaluations between different populations, contributing to the growth and planning of health policies [48]. In this sense, the results of our study showed that boys scored significantly higher than girls with boys showing a significantly higher level of PA than girls. The differences in the level of PA observed in boys and girls have been detected in different studies [11,38,49]. This may be due to the intensification of academic work, which seems to affect adolescent girls more than boys. In this sense, when there is a certain confrontation between studies and physical and sporting activity for girls, they tend to reduce their level of PA or directly abandon it [50]. Likewise, young girls highlight the barrier of not having anyone to practise with, which underlines the social component of the difficulties in practising at this age [51].

Few studies in Peru have evaluated the level of PA in adolescents but in this regard, the research of Cossio-Bolaños et al. [52] agreed with the results of this study, reporting that boys showed higher PA values than girls as assessed by a PA level questionnaire.

However, a study was found in Colombia where the results showed that girls obtained higher values in vigorous and moderate PA compared with boys [53]. This may be due to the fact that this study included a wider age range (10–20 years) than the studies mentioned above.

Regarding the BMI results of our study, we observed that the higher the weight category, the lower the PA level. This could be explained by the inverse correlation between the BMI and PA level in adolescents [53]. Similar results were obtained in other studies [54,55,56]. 

Therefore, the promotion of PA, with its respective improvement of PF, is indispensable as a tool for the prevention of being overweight and obesity as it has also been observed that as the BMI increases, the level of quality of life decreases in adolescents [57].

Regarding the self-perception of PF, the results showed that boys presented a better self-perception of PF compared with girls with the exception of flexibility. Similarly, Ortega et al. indicated that boys obtained a higher level of physical self-perception compared with girls with the exception of flexibility [42], also reported by Cossio-Bolaños [52], García-Rubio et al. [49] and Castro-Sánchez et al. [58]. The lower self-perception of PF in girls may be due to the fact that during adolescence, statistically significant differences appear in the physical self-concept according to sex with males presenting better self-perceptions in all dimensions, results that appear repeatedly in previous studies [59,60,61].

The main findings of our study indicated that there was a direct correlation between the level of PA in adolescents and their self-perceived PF. Therefore, adolescents who practiced more PA were perceived to have a better overall PF, as reported by other studies that obtained this relationship [62,63,64]. Along the same lines, this relationship was found in Colombian university students [65] as well as in the adult population [66,67,68].

Therefore, the perception that adolescents have of their self-perceived PF could have a direct relationship with the practice of more or less PA. However, one of the limitations of this study was that, due to its cross-sectional nature, causal relationships could not be established. This was because the level of PA was assessed on the basis of a self-reported questionnaire, which did not allow us to establish an objective level of PA of the adolescents. In this sense, future studies could consider other more objective methods for the assessment of PA in addition to the relationship of PA with self-perceived health status, life satisfaction, health-related quality of life, education, culture or environment. Furthermore, it has been suggested that improvements in general physical fitness may have a favourable effect on positive perceptions of health. Therefore, this will undoubtedly have a positive impact on the present and future health and wellbeing of adolescents. Finally, it should be noted that the specificity of the study in Peru and the results reported for it may be relevant as the particularities of each country in terms of socio-demographics such as socio-economic status or cultural variables may be influential in obtaining one result or another. Therefore, these results could lead to the orientation of appropriate and effective prevention and intervention programmes and strategies. 

## 5. Conclusions

The present study found a direct relationship between the level of PA and self-perceived PF. Furthermore, based on the results obtained, it was shown that Peruvian boys had a higher level of PA than girls. This gender difference was also repeated with respect to self-perceived PF with the exception of flexibility where the highest results were found in Peruvian girls. It was concluded that the higher the weight category, the lower the level of PA. In this sense, it is recommended that all those professionals who contribute to improving the health of adolescents (doctors, scientists, PE teachers, etc.) promote measures for greater adherence to PA in girls as well as measures to prevent sedentary lifestyles and physical inactivity in order to combat excess body fat at an early age.

## Figures and Tables

**Table 1:** Socio-demographic comparison of participants.

	Males (*n* = 607)	Females (*n* = 622)	Total (*n* = 1229)	*p*-Value	Cohen’s d
Anthropometric Characteristics					
Age (years)	14.53 (1.71)	14.56 (1.72)	14.54 (1.71)	0.795	0.020
Weight (kg)	54.19 (7.78)	52.75 (7.49)	53.46 (7.66)	0.002	0.188
Height (cm)	155.0 (11.85)	150.5 (8.93)	152.8 (10.7)	<0.001	0.429
IMC (kg/m^2^)	22.55 (2.25)	23.25 (2.54)	22.90 (2.43)	<0.001	−0.291
PAQ-A (score)	2.41 (0.58)	2.27 (0.57)	2.34 (0.58)	<0.001	0.121
IFIS					
General Fitness Status (Score)	3.13 (0.619)	2.97 (0.731)	3.07 (0.685)	<0.001	0.236
Very Good (%)	0.8	0.4	2.4		
Good (%)	9.6	16.4	13.0		
Acceptable (%)	61.4	58.6	60.0		
Bad (%)	28.2	21.0	24.6		
Very Bad (%)	0	0	0		
Cardiorespiratory Fitness (Score)	3.28 (0.770)	3.07 (0.766)	3.17 (0.775)	<0.001	0.273
Very Good (%)	0	0	0		
Good (%)	10.7	19.6	15.2		
Acceptable (%)	59.9	60.7	60.3		
Bad (%)	20.5	13.2	16.8		
Very Bad (%)	8.9	6.6	7.7		
Muscular Strength (Score)	2.99 (0.858)	2.72 (0.874)	2.85 (0.876)	<0.001	0.312
Very Good (%)	4.1	6.4	5.3		
Good (%)	24.9	36.9	31.0		
Acceptable (%)	38.9	35.5	37.2		
Bad (%)	32.0	21.0	26.4		
Very Bad (%)	0	0.2	0.1		
Speed Agility (Score)	3.29 (0.931)	3.06 (0.958)	3.17 (0.951)	<0.001	0.243
Very Good (%)	0	0	0		
Good (%)	20.5	31.5	26.0		
Acceptable (%)	42.9	42.7	42.8		
Bad (%)	24.1	14.3	19.1		
Very Bad (%)	12.5	11.6	12.0		
Flexibility (Score)	3.06 (0.737)	3.04 (0.709)	3.05 (0.723)	0.585	0.028
Very Good (%)	1.8	0.8	1.3		
Good (%)	15.7	17.3	16.5		
Acceptable (%)	60.7	61.6	61.2		
Bad (%)	18.5	17.0	17.7		
Very Bad (%)	3.3	3.2	3.3		

*p*-value: sex differences were analysed using the Mann–Whitney U test for non-parametric variables.

**Table 2:** PAQ-A population normative values by gender, age and BMI categories.

			PAQ-A Utility Index									
	Male	Female	Male			Female						
	N (%)	N (%)	Mean	SD	Median	RI	Mean	SD	Median	RI	*p*	Cohen’s d
Age												
12	99 (16.3)	101 (16.2)	2.43	0.484	2.43	0.645	2.30	0.605	2.17	0.784	0.023	0.237
13	101 (16.6)	100 (16.1)	2.45	0.5440	2.42	0.621	2.30	0.498	2.30	0.596	0.033	0.288
14	100 (16.3)	100 (16.1)	2.30	0.619	2.17	0.817	2.22	0.517	2.15	0.563	0.372	0.140
15	100 (16.5)	100 (16.1)	2.51	0.666	2.53	0.979	2.23	0.588	2.09	0.785	0.002	0.446
16	100 (16.5)	112 (18.0)	2.38	0.619	2.32	0.845	2.35	0.661	2.17	0.803	0.448	0.046
17	108 (17.8)	109 (17.5)	2.41	0.537	2.42	0.651	2.22	0.576	2.05	0.628	0.003	0.341
IMC Category												
Normal Weight	289 (47.6)	298 (47.9)	2.45	0.578	2.42	0.776	2.33	0.579	2.17	0.755	0.003	0.044
Overweight	235 (38.7)	267 (42.9)	2.40	0.571	2.40	0.713	2.26	0.554	2.17	0.672	0.001	0.249
Obese	79 (13.0)	55 (8.8)	2.28	0.545	2.29	0.674	1.95	0.514	1.80	0.770	<0.001	0.622

**Table 3:** Correlation between the level of physical activity and self-perceived level of physical fitness.

		General Fitness Status	Cardiorespiratory Fitness	Muscular Strength	Speed Agility	Flexibility
PAQ-A	All Participants	0.644 *	0.638 *	0.604 *	0.613 *	0.404 *
	Male	0.594 *	0.563 *	0.580 *	0.565 *	0.336 *
	Female	0.673 *	0.692 *	0.594 *	0.633 *	0. 466 *

* Significative at level *p* < 0.012.

## Data Availability

The datasets used during the current study are available from the corresponding author on reasonable request.
